# Surface properties of MoS_2_ probed by inverse gas chromatography and their impact on electrocatalytic properties

**DOI:** 10.1039/c7nr07342a

**Published:** 2017-11-13

**Authors:** Eva Otyepková, Petr Lazar, Jan Luxa, Karel Berka, Klára Čépe, Zdeněk Sofer, Martin Pumera, Michal Otyepka

**Affiliations:** a Regional Centre of Advanced Technologies and Materials , Department of Physical Chemistry , Palacký University Olomouc , tř. 17. listopadu 12 , 771 46 Olomouc , Czech Republic . Email: Michal.Otyepka@upol.cz; b Department of Inorganic Chemistry , University of Chemistry and Technology , 166 28 Prague 6 , Czech Republic; c Division of Chemistry & Biological Chemistry , School of Physical and Mathematical Sciences , Nanyang Technological University , Singapore , 637371 , Singapore . Email: pumera.research@gmail.com

## Abstract

Layered transition metal dichalcogenides (TMDs) are at the forefront of materials research. One of the most important applications of these materials is their electrocatalytic activity towards hydrogen evolution, and these materials are suggested to replace scarce platinum. Whilst there are significant efforts towards this goal, there are various reports of electrocatalysis of MoS_2_ (which is the most commonly tested TMD) with large variations of the reported electrocatalytic effect of the material, with overpotential varying by several hundreds of millivolts. Here, we analyzed surface properties of various bulk as well as single layer MoS_2_ samples using inverse gas chromatography. All samples displayed significant variations in surface energies and their heterogeneities. The surface energy ranged from 50 to 120 mJ m^–2^ depending on the sample and surface coverage. We correlated the surface properties and previously reported structural features of MoS_2_ with their electrochemical activities. We concluded that the observed differences in electrochemistry are caused by the surface properties. This is an important finding with an enormous impact on the whole field of electrocatalysis of layered materials.

## Abstract 2

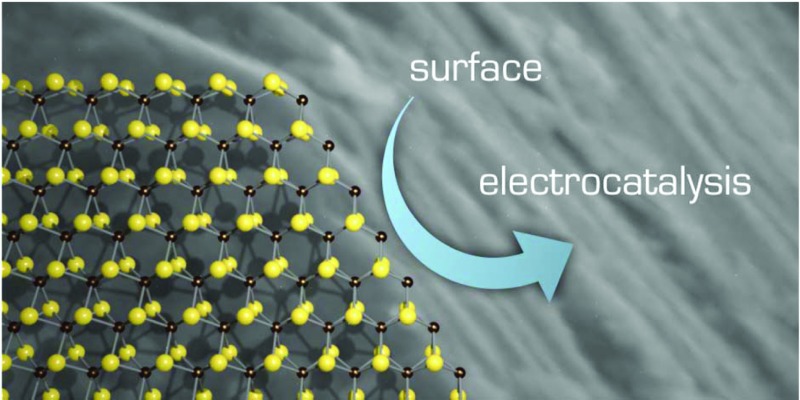
Differences in the electrochemistry of MoS_2_ samples are caused by their surface properties.

## Introduction

Molybdenum disulfide (MoS_2_) is an archetypal member of the family of transition metal dichalcogenides (TMDs) that are undergoing a scientific renaissance because of their layered structure and the possibility of isolation of individual sheets as formally two-dimensional (2D) materials. MoS_2_ is a chemically and thermally stable material, which is used as a dry lubricant,^1^ catalyst for hydrogenation^2^ and hydrodesulfurization.^3^ Bulk MoS_2_ is an indirect semiconductor whereas single layer MoS_2_ has a direct band gap of 1.8 eV.^4^–^6^ These properties make MoS_2_ a promising material for the construction of novel electronic devices, *e.g.*, memristors,^7^ photodetectors^8^ and interband tunnel field effect transistors.^9^

MoS_2_ was proposed as a substitute of precious platinum catalysts for the hydrogen evolution reaction (HER). The HER is a thoroughly investigated reaction that has gained renewed attention in recent years since hydrogen plays a key role as an energy carrier in future green technologies. The HER catalytic activity of MoS_2_ was first predicted by density functional theory calculation,^10^ which then stimulated a number of experimental studies showing that MoS_2_ had better activity than most of the non-precious metals.^11^ It was also revealed that the edge states at the MoS_2_ edges were responsible for its catalytic activity, while the basal plane was inert and insulating.^12^ There is, however, a wide range of overpotentials and Tafel slopes reported for various MoS_2_-based catalysts. Recently, Chua *et al.* tested seven commercially available MoS_2_ samples and observed a large variance of their catalytic properties.^13^ Their HER performance was only modest, which was attributed to low phase purity. An optimal MoS_2_ electrocatalyst should combine an efficient electrical contact with a plentitude of catalytically active edge sites. However, a suitable method for surface characterization and, in particular, an estimation of the amount of edge sites in a large amount of samples is still missing. The need for a such method is not trivial, as overpotentials of the HER at MoS_2_ reported in the literature vary by 500 mV and the reason for such a discrepancy is still unclear.^13^

Inverse gas chromatography (iGC) is a surface characterization technique, which was pioneered in 1941 and matured into a very robust tool providing very useful information about surface nature, heterogeneity *etc*.^14^–^16^ iGC provides averaged information about the complete surface, because it is based on the interaction of gas probes, which flow through the bed made of studied materials. The adsorption/desorption events result from probe–surface interactions and determine the retention of the probe by the sample. From the retention times of probes, information about the sample free energy and its dispersive and specific acid–base components can be derived (see the Experimental section for details). The probe preferentially adsorbs to sites with high probe–sample affinities, *i.e.*, to so-called high-energy sites. In our previous study, we utilized this feature to assess the amount of surface high-energy sites and the surface homogeneity of graphene nanopowder.^17^ We found that the high-energy sites were the edges and surface steps by comparing measured adsorption enthalpies of acetone molecules at low coverage to those calculated using density functional theory (DFT). Ferguson *et al.* adopted the same approach, *i.e.*, the finite-dilution iGC, to show that the dispersive component of the surface energy of graphite had contributions from edge and basal plane defects as well as from the hexagonal carbon lattice.^18^

Here, we analyze the surface properties of eight bulk MoS_2_ samples from various providers and two exfoliated MoS_2_ by the iGC method. We utilize one of the advantages of the method – that one can control the coverage of probe molecules on the surface by controlling the probe injection time. We determined the total surface free energies and their dispersive and acid–base components at surface coverage ranging from 1 to 20% of the monolayer. We realized that the individual samples differ significantly in their surface properties, namely, surface energies and surface heterogeneity. We also related the acquired data with electrochemical experiments, which sampled the studied materials for the HER and heterogeneous electron transfer, *i.e.*, applications in electrocatalysis and electrochemical sensing. Our work shows that there is a correlation between the electrochemical activity and the adsorption energy at high-energy sites, content of oxygen, and specific surface area.

## Results and discussion

### iGC surface energy measurements of bulk MoS_2_

**Fig. 1:**
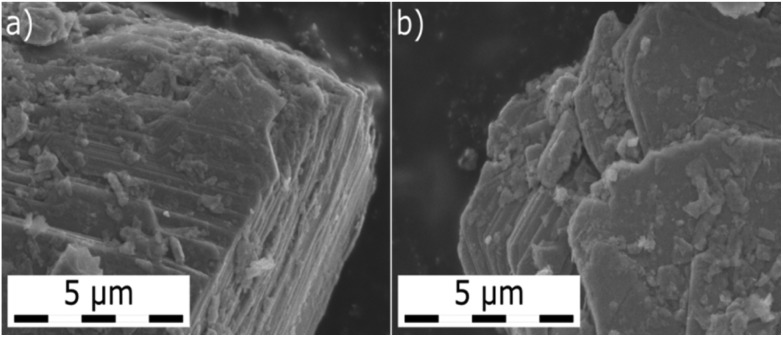
Two images of the MoS_2_ sample from Strem acquired by scanning electron microscopy (SEM) show the layered and flaky nature of the MoS_2_. Structural features of this layered material, *i.e.*, edges, steps and terraces, which represent the high-energy sites, can be well recognized.

**Fig. 2:**
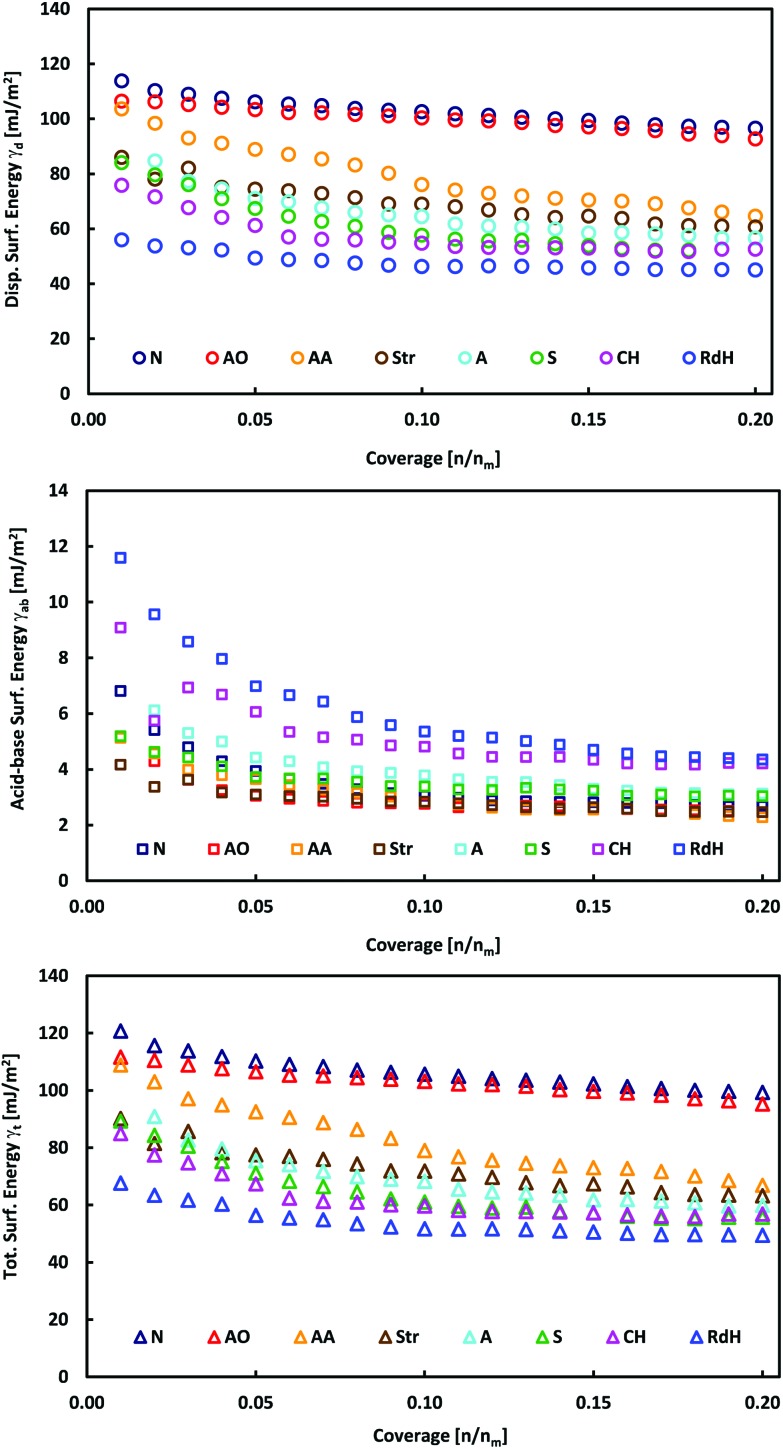
Plots of total, *γ*_t_, dispersive, *γ*_d_, and acid–base, *γ*_ab_, surface energies as a function of surface coverage for bulk MoS_2_ samples.

The individual MoS_2_ samples from various providers display a layered structure with a significant amount of edges, steps and terraces, which can be recognized in their SEM images (Fig. 1 and Fig. 1 in ref. 13). iGC experiments showed that they differ in their dispersive, acid–base and total surface free energies. In all cases, the total surface free energies depend on the surface coverage (Fig. 2). The *γ vs.* coverage curves have a slightly concave shape, with surface energy falling with increasing probe coverage. At the low surface coverage of 1%, the surface energies range from 121 (Natural) to 68 mJ m^–2^ (Riedel de Haën). At the higher surface coverage of 20% of the monolayer, they reach from 99 (Natural) to 49 mJ m^–2^ (Riedel de Haën). Both acid–base and dispersive parts of the surface energy drop with surface coverage (Fig. 2). Generally, the samples maintain their order according to the total free surface energies in the whole studied range of surface coverage, only with one exception (Aldrich). The decrease of the surface energy with coverage is caused by the fact that at the low coverage, the probes adsorb at the sites with high surface energy (high-energy sites). Surface irregularities, *e.g.*, steps, edges, and cavities, represent the typical high-energy sites in layered van der Waals (vdW) materials (*cf.*, Fig. 1).^17^,^19^,^20^ The surface energies measured at low coverage are a result of the intermixing of adsorption on high-energy sites and basal plane adsorption.^17^ The amount of high-energy sites is rather low, which is indicated by only a slightly concave shape of the surface energy, particularly in comparison with analogical iGC measurements on graphite.^17^,^18^

These findings also imply that the surface energies probed at low coverage on the sample surface are not representative of the surface as a whole. As soon as the high-energies become occupied, the probes adsorb to the bare surface^17^ (basal plane/terraces, *cf*. Fig. 1) and the surface free energy becomes constant. Taking these facts into account, we may deduce that the Natural and Acros Organics samples have the highest amount of high-energy sites, whose surface energy is equal to ∼100–120 mJ m^–2^. On the other hand, the surface energy values of ∼50–100 mJ m^–2^ measured at 20% surface coverage represent the surface energy of the MoS_2_ basal plane surface. It should be mentioned that although it is possible to measure at even higher surface coverage, we expect that mutual interactions (clustering) of probe molecules at high coverage would spoil the measured surface energy.^21^

### Basal plane surface energy of MoS_2_

In order to corroborate that the values measured at 20% coverage are indeed the basal plane surface energies of individual samples, we compared measured surface energies to literature values. In 1988, Kelebek reported molybdenite to be hydrophobic with a critical surface tension of 29 mJ m^–2^.^22^ Coleman *et al.*^23^ studied the dispersion and exfoliation of transition metal dichalcogenides MoS_2_ WS_2_, MoSe_2_, and MoTe_2_, and found that they have surface energies of 70–75 mJ m^–2^. Gaur *et al.*^24^ examined a layer thickness-dependent wettability of MoS_2_ and concluded that the lower the thickness is, the higher the contact angle will be. They obtained the surface energy of few layer MoS_2_ as 44.5 mJ m^–2^ (Neumann method) and 40.5 mJ m^–2^ (Fowkes method). The discrepancy between the surface energy values can be attributed to different methods used to calculate surface energy from wetting experiments and to spontaneous contamination by ambient airborne contaminants. Kozbial and coworkers^25^ have recently shown that the clean surface of bulk MoS_2_ is intrinsically mildly hydrophilic with a water contact angle of 69.0° and a surface energy of 54.5 mJ m^–2^. Upon exposure to ambient air for one day, the MoS_2_ surface adsorbs hydrocarbons from the surrounding environment, reducing the apparent surface energy. Therefore, our values in the range of 50–100 mJ m^–2^ (measured at 20% coverage) agree with previous experiments and represent the surface energy of the MoS_2_ basal plane surface.

Furthermore, theoretical calculations of the surface energy may provide independent and valuable insight, since they can evaluate the surface energy of perfect and clean surfaces. However, in layered materials, the surface energy and closely related binding energy are dominated by vdW interactions. Unfortunately, widely used local and semilocal exchange and correlation functionals (local density approximation and generalized gradient approximation) fail to account for nonlocal electron–electron correlation effects, which significantly contribute to vdW forces. The adiabatic-connection fluctuation-dissipation theorem within random phase approximation (ACFDT-RPA) is believed to be the most accurate of commonly used methods, but it is limited to small systems due to its computational complexity.^26^,^27^ The ACFDT-RPA approach was applied to MoS_2_ and other dichalcogenides by Björkman *et al.*,^28^ who obtained a binding energy of 20.5 meV Å^–1^ for MoS_2_, which corresponded to the surface energy of 164 mJ m^–2^. This value is higher than that obtained from experiments. This discrepancy can be explained by airborne surface contamination, which should lower the observed surface energies, particularly in the case of contact angle measurements. It should be noted that theoretical surface energies higher than the experimental values were also reported for graphite and graphite fluoride.^29^–^31^ Our iGC approach should be less sensitive to airborne contaminants at the surface, because carrier gas flow used during the experiment should wipe out most of the physisorbed contaminants. Indeed, the surface energies measured by iGC are in general higher than their contact angle counterparts measured on the same kind of material (graphite, graphite fluoride, MoS_2_*etc*.).

In all cases, the total surface free energy is dominated by the dispersive part, which contributes on average 94% to the total surface energy (Table 1). This is in excellent agreement with ACFDT-RPA calculations by Bjorkman *et al.*,^28^ which revealed that the interlayer binding of MoS_2_ is dominated by vdW interactions. The electrostatic and covalent interaction contributed little to interlayer binding. The acid–base contribution is higher at low surface coverage amounting to 8% and decreases with coverage to 5% (Fig. 2 and Table 2). The acid–base component may arise from the interaction of the probes with polar contaminating functional groups, *e.g.*, emerging from oxidation. Such contaminant groups are preferentially present at the high-energy sites, *i.e.*, at surface structural defects like edges, steps, and cavities.

**Table 1:** Total *γ*_t_, dispersive *γ*_d_, and acid–base *γ*_ab_, surface free energies and contribution of dispersive surface free energy to the total surface free energy at 1% (indexed as max) and 20% (indexed as min) surface coverage values. *p*_1_, *p*_2_, *p*_3_ parameters of the fitting of *γ*_t_ as *f*(*ν*) (see the Experimental section for details) are listed with their confidence interval at α = 0.05. *R*^2^ is the coefficient of determination

Sample	*γ*_t,max_, mJ m^–2^	*γ*_t,min_, mJ m^–2^	*γ*_d,max_, mJ m^–2^	*γ*_d,min_, mJ m^–2^	*γ*_ab,max_, mJ m^–2^	*γ*_ab,min_, mJ m^–2^	*γ*_d,max_/*γ*_t,max_, %	*γ*_d,min_/*γ*_t,min_, %	*p*_1_, mJ m^–2^	*p*_2_	*p*_3_, mJ m^–2^	*R* ^2^
Alfa Aesar	109	67	104	65	5.1	2.3	95	97	52 ± 4	0.09 ± 0.02	60 ± 5	0.9905
Acros Organics	111	95	106	93	5.1	2.5	95	98	43 ± 32	0.02 ± 0.02	69 ± 32	0.9878
Aldrich	91	60	85	56	6.2	3.1	93	93	38 ± 3	0.13 ± 0.03	57 ± 3	0.9789
Chempur	85	56	76	52	9.1	4.2	89	93	37 ± 2	0.25 ± 0.03	56 ± 1	0.9912
Natural	121	99	114	97	6.8	2.7	94	98	24 ± 2	0.11 ± 0.03	97 ± 2	0.9858
Riedel de Haën	68	49	56	45	11.6	4.3	82	92	22 ± 1	0.20 ± 0.02	49 ± 1	0.9912
Schuchard	89	55	84	52	5.2	3.0	94	95	43 ± 1	0.18 ± 0.01	54 ± 1	0.9971
Strem	90	63	86	61	4.2	2.5	95	97	33 ± 5	0.09 ± 0.04	58 ± 7	0.9635
NaNAFT	102	62	92	57	10.6	5.6	90	92	54 ± 11	0.22 ± 0.07	62 ± 3	0.9467
BuLi	100	58	93	54	9.0	4.8	93	93	57 ± 4	0.22 ± 0.03	57 ± 2	0.9847

**Table 2:** List of analyzed MoS_2_ samples, their mass used in the experiment, N_2_ specific surface area (SSA), formula (from XPS), content of oxygen (from XPS), arithmetic diameter from laser diffraction (*D*), content of the 2H phase, OP is the overpotential, TS is the Tafel slope, PS is peak separation and pECD is –log_10_ of exchange current density (in mA cm^–2^)

Sample	Label	Mass, mg	SSA, m^2^ g^–1^	MoS_*x*_^*a*^	MoO_*x*_	*D*, μm	2*H* cont., %	OP, V	TS, mV dec^–1^	PS, mV	pECD
Acros Organics	AO	105.8	6.30^*a*^	2.11^*a*^	0.31	4^*a*^	100^*a*^	0.587^*a*^	132^*a*^	98	1.951
Aldrich	A	57.8	10.37^*a*^	2.24^*a*^	0.26	3^*a*^	100^*a*^	0.603^*a*^	149^*a*^	81	1.920
Alfa Aesar	AA	196.5	3.64^*a*^	2.41^*a*^	0.13	24^*a*^	58^*a*^	0.690^*a*^	173^*a*^	65	3.473
Chempur	CH	128.9	4.81^*a*^	2.33^*a*^		6^*a*^	100^*a*^	0.725^*a*^	161^*a*^	101	3.884
Natural	N	390.5	1.25^*a*^	2.29^*a*^	0.12	44^*a*^	100^*a*^	0.660^*a*^	171^*a*^	124	3.016
Riedel de Haën	RdH	395.5	1.57^*a*^	2.49^*a*^	0.22	15^*a*^	57^*a*^	0.779^*a*^	163^*a*^	120	4.369
Schuchard	S	121.2	3.93^*a*^	2.24^*a*^	0.47	5^*a*^	62^*a*^	0.664^*a*^	152^*a*^	105	3.108
Strem	Str	182.8	3.28	2.18	0.15	24	100	0.714	172	103	3.294
NaNAFT	NaNAFT	37.2	11.5	2.03	1.73		100	0.467	84	97	2.949
BuLi	BuLi	79.3	6.86	2.10	0.49		100	0.618	150	93	1.974

^*a*^Adopted from ref. 13.

### iGC surface energy measurements of exfoliated MoS_2_

**Fig. 3:**
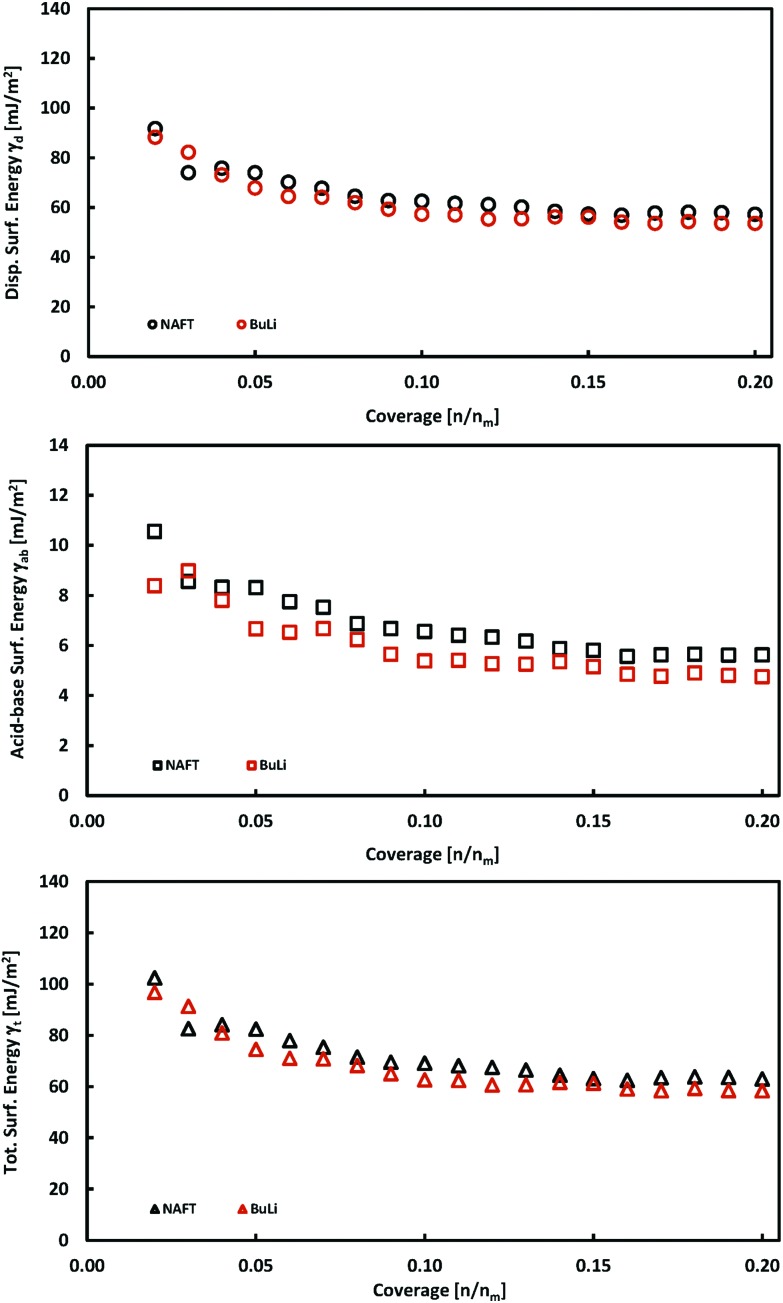
Plots of total, *γ*_t_, dispersive, *γ*_d_, and acid–base, *γ*_ab_, surface energies as a function of surface coverage for exfoliated MoS_2_ samples.

The exfoliation of bulk MoS_2_ leads to few-layered materials, which have a higher surface area and have higher catalytic activities, because the exfoliation process increases the concentration of edge sites which are catalytically active. We exfoliated MoS_2_ using two exfoliation agents: *n*-butyllithium (BuLi) and sodium naphthalenide (NaNAFT). Both these reagents are highly efficient and sodium naphthalenide can be used for the high yield preparation of single-layer thin MoS_2_. The NaNAFT sample shows the highest surface area among the studied samples (Table 1). We analyzed the surface energy of both the exfoliated samples using the iGC technique and the acquired properties, which are similar to those observed for bulk MoS_2_ samples. Both samples display very similar surface characteristics having the total surface free energies at a low coverage of ∼100 mJ m^–2^, which drop to ∼60 mJ m^–2^ at 20% surface coverage (Fig. 3). The exfoliated MoS_2_ is prone to oxidation,^32^ which is also reflected by the highest content of oxygen in the exfoliated samples (Table 2). One would expect that the highest content of oxygen in the NaNAFT sample could increase the acid–base contribution to the total surface energy, which was not, however, reflected in the data. To sum up this part, we may conclude that the exfoliation of MoS_2_ does not significantly change the surface properties of this transition metal dichalcogenide.

### Correlation to electrochemical properties

Our iGC data (Fig. 2 and 3) show that the surface energy is high at low surface coverage and falls with increasing coverage due to the presence of the high-energy adsorption sites. Since the high-energy sites are predominantly edges and surface steps, *i.e.*, the features which are active catalytic sites for the HER, one may wonder how are their abundance and surface energy related to the electrochemical performance of the samples.

The electrochemical properties of the samples were determined as described in the Experimental section. Indeed, their catalytic properties (the overpotential and Tafel slope, ranging from 0.58 to 0.78 V *vs.* RHE and from 132 to 173 mV dec^–1^, respectively) were inferior to the properties of single layer MoS_2_ samples. MoS_2_ nanoplatelets on the Au[111] support showed an onset at ∼–0.15 V *vs.* RHE, and a Tafel slope of 55–60 mV dec^–1^.^12^ MoS_2_ on porous carbon paper has similar properties to the gold supported nanoplatelets, with an onset at ∼–0.2 V *vs.* RHE and a Tafel slope of 120 mV dec^–1^.^33^

**Fig. 4:**
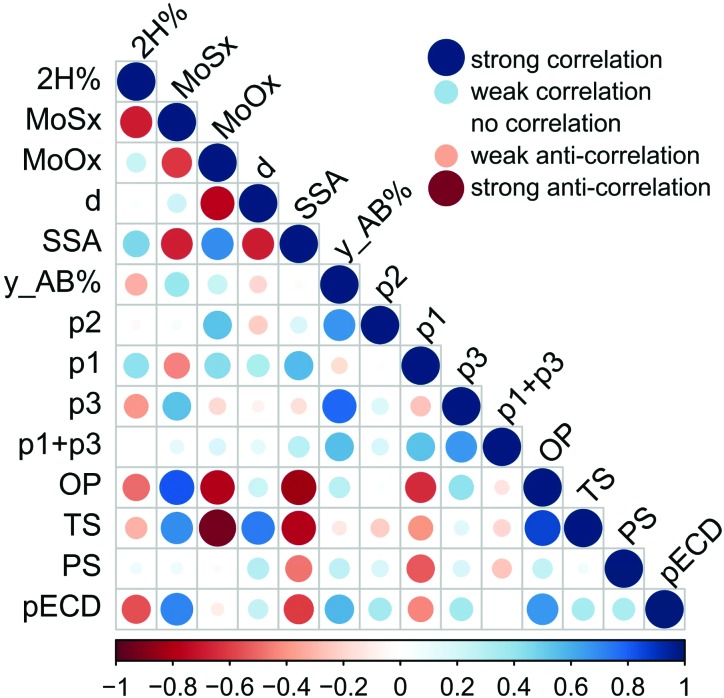
Correlation plot of structural, surface and electrochemical properties of all MoS_2_ samples. The size and the color of the dot correspond to the value of the correlation coefficient, which is shown on the scale bar below the plot. 2H% stands for the percentage of the 2H phase, MoS_*x*_ and MoO_*x*_ for contents of sulfur and oxygen, respectively, *d* is the arithmetic diameter, SSA is the specific surface area, y_AB% is the percentage of acid–base contribution to the total surface energy, *p*1–*p*3 are the fitting parameters explained in the methods, OP is the overpotential, TS is the Tafel slope, PS is peak separation and pECD is the log_10_ of exchange current density.

We correlate electrochemical and electrocatalytic properties of individual MoS_2_ samples with their surface and structural properties. Specifically, we examine the correlation between the electrochemical performance (the Tafel slope, overpotential, and exchange current density) and sample surface characteristics such as the specific surface area, surface energy at low coverage (infinite dilution limit), and the *p*_2_ parameter expressing the relative amount of high-energy sites. Furthermore, we inspect the correlation to the oxygen content in the samples, as the edges of MoS_2_ are susceptible to oxidation, and the sulfur content, because only edges having specific sulfur coverage are active for the HER.^34^ The correlations are displayed in Fig. 4. One can identify that both the overpotential and the Tafel slope have significant negative correlation to the specific surface area and to the content of oxygen. The correlation to the specific surface area can be understood by noting that the number of edge HER active sites scales with the flake size, and, consequently, with the specific surface area. The negative correlation to the oxygen content in the samples is less intuitive. A recent study showed the susceptibility of the edges of CVD grown MoS_2_ samples to oxidation when stored in air for a longer period.^35^ Our consequent theoretical study^32^ has revealed that the oxidation proceeds because of the low energetic barrier to the dissociation of molecular oxygen. The barrier at the edge is much lower than that on the MoS_2_ surface, making edges and grain-boundaries prominent sites for oxidation. Indeed, the amount of oxygen has a positive correlation with both the specific surface area and the relative amount of high-energy sites *p*_2_.

Furthermore, both the overpotential and the Tafel slope display a negative correlation with the *p*_1_ parameter, which corresponds effectively to the adsorption energy to high-energy sites. There seems to be a clear logic behind this observation because the only edges of MoS_2_ are active for the HER^12^ and, at the same time, the edges and surface steps are usual high-energy sites for adsorption. Yet, the HER is a complex reaction involving the bonding of hydrogen on a catalyst followed by the release of molecular hydrogen, and only edges having specific sulfur coverage are active for the HER.^34^ Much larger molecules (*n*-alkanes) are being used as probes to measure the free energy of adsorption (see the Experimental section), and the characteristics of high-energy sites for these molecules must not coincide with that of the sites efficient for the HER, because the adsorption sites are gas probe sensitive.^20^ This issue shall be addressed in future studies.

Overall, our analysis shows that the electrochemical and electrocatalytic properties are related to several structural and surface features of MoS_2_ and it is likely that these features are mutually related and contribute in synergy to the HER catalytic efficiency of a material. This idea can be supported by the fact that we were able to construct multilinear fits of electrochemical properties *vs.* structural and surface features (involving only two variables from structural and surface features) having high coefficients of determinacy (*r*^2^ > 0.9); however, due to the limited number of data points, we would prefer not to draw any strong conclusion from such relationships.

## Conclusions

We have provided an in-depth analysis of the hydrogen evolution reaction on layered TMDs and its correlation to surface adsorption properties. We analyzed the surface properties of bulk MoS_2_ materials by the iGC method. We determined total surface free energies and their dispersive and acid–base components at surface coverage ranging from 1 to 20% of the monolayer. We found that individual MoS_2_ samples from various providers differed significantly in their surface energy and surface heterogeneity. In all cases, the total surface free energies depended on the surface coverage and the resulting curves had a concave shape, with surface energy decreasing with increasing probe coverage. The decrease of the surface energy with coverage occurred because, at low coverage, the probes adsorbed at sites with high surface energy (high-energy sites) such as edges, cavities, and surface steps. As soon as the high energy sites became occupied, the probes adsorbed to the basal plane surface and the surface free energy became constant. We obtained the basal plane surface energy in the range of 50–100 mJ m^–2^ for samples from various providers. Upon correlating the surface properties to the electrochemical data, we found that the overpotential and the Tafel slope had significant negative correlation to the content of oxygen, the specific surface area, and adsorption energy at high-energy sites. Our results suggested that surface features and properties were mutually related and contributed in synergy to the HER catalytic efficiency of selected materials. Our work provided deep insight into the correlation of the surface energy of MoS_2_ and its electrochemistry and it shall have a profound practical impact on the characterization of a large amount of powder MoS_2_ (and transition metal dichalcogenides in general) and its suitability for electrocatalysis.

## Experimental

### Theory of surface energy analysis

In the iGC experiment, the known probe solvents are injected into a silanized glass column and flow through the sample bed.

The concentration of solvent transmitted through the column is recorded as a function of time in the form of a chromatogram. The adsorption–desorption behavior of the probe on the solid surface is derived from the retention time, *t*_r_, which is the time taken for the probe to go through the column. The retention time is used to calculate the net retention volume, *V*_N_, which is a fundamental thermodynamic property of solid–vapor interactions, using eqn (1):1
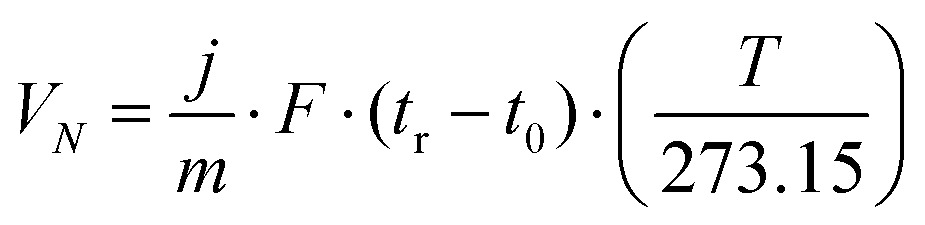
where *j* is the James–Martin correction factor for pressure drop, *m* is the mass of the sample in the column, *F* is the carrier gas flow rate, *t*_0_ is the dead time (time taken for the noninteracting probe, *e.g.*, methane, to go through the column), and *T* is the column temperature. We can further use the *V*_N_ values to calculate the surface energy. The surface energy of a material, *γ*, consists of two components, the dispersive (*γ*_d_) and acid–base surface energies (*γ*_ab_), *i.e.*, *γ* = *γ*_d_ + *γ*_ab_. The dispersive surface energy of a material stems namely from the London (dispersion) interactions. The Schultz and Dorris & Gray methods are widely used for calculating *γ*_d_ using iGC and they provide similar results at ambient temperatures. On the other hand, the Dorris & Gray method is temperature corrected and should be used for higher temperature experiments, which was also our case. It should be noted that the Dorris & Gray method was initially developed for infinite dilution systems. But it has become standard practice to apply the Dorris & Gray method to finite dilution experiments. In the Dorris & Gray method, a series of *n*-alkanes are used as probes to measure the free energy of adsorption. The dispersive free energy of one methylene group (Δ*G*_CH_2__) can be calculated from the slope of the alkane line by plotting probe adsorption free energies *versus* the carbon number, *n*, of the alkane probe using eqn (2):2
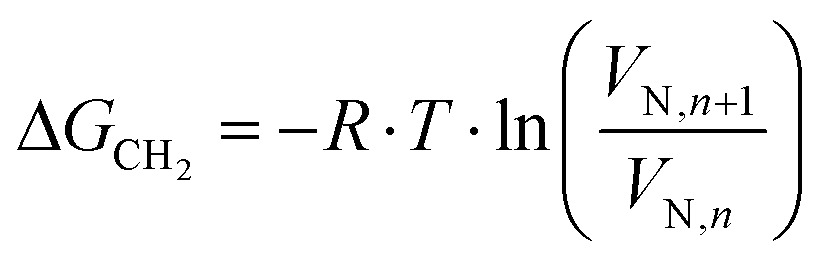
where *T* is the column temperature and *V*_N_ is the net retention volume. Δ*G*_CH_2__ is related to the work of adhesion of the methylene group, *W*_CH_2__, which can be calculated by using eqn (3):

3 Δ*G*_CH_2__ = –*N*_a_·*a*_CH_2__·W_CH_2__

where *N*_a_ is the Avogadro's number, *a*_CH_2__ is the cross-sectional area of an adsorbed methylene group. Using the Fowkes relation, the work of adhesion of the methylene group is a geometric mean of the dispersive free surface energy and the dispersive surface energy of a methylene group (eqn (4)):4
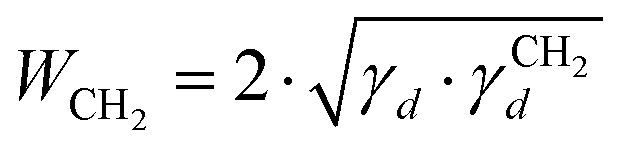
where 
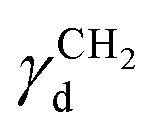
 is the dispersive surface energy of a methylene group, which is calculated as 
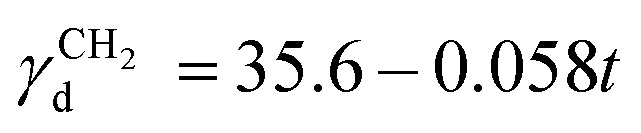
, where *t* is the temperature in °C.^36^ Combining eqn (2)–(4), we obtain an equation for *γ*_*d*,*ν*_ (eqn (5)), the isosteric dispersive surface energy of the solid sample:5
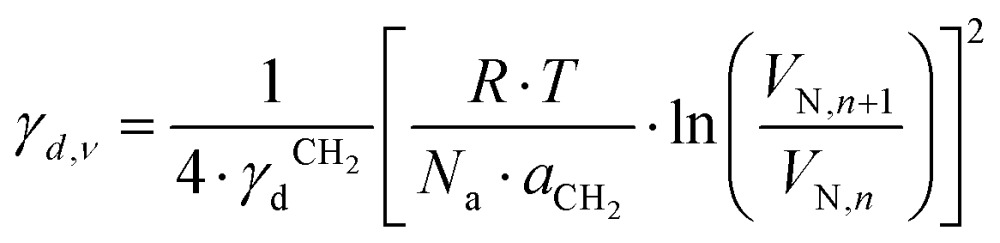



The retention volume is measured for a set of alkane probes and the isosteric surface energy calculated at the given surface coverage, *ν*, using eqn (5). This is repeated in a range of coverage values to produce a surface energy profile *vs.* coverage.

The acid–base component of the surface energy *γ*_ab_ is associated with the specific interactions between a probe and the surface, *e.g.*, hydrogen bonding, and it was determined using the van Oss–Good–Chaudhury approach^37^ with the Della Volpe scale.^38^ Dichloromethane was used as a monopolar acid probe and ethylacetate was used as a monopolar basic probe to characterize the basic and acidic characteristics of the solid surface.^39^ It should be noted that the absolute values of the estimated acid–base components should be interpreted with care and can be preferentially used for relative comparisons.^14^

The measured *γ*_*t*_ was fitted as a function of coverage *f*(*ν*) using eqn (6), using nonlinear regression by the least-squares method as implemented in a statistical software QC-Expert 3.3

6 *γ*_*t*_ = *p*_1_·exp(–*p*_2_·*ν*) + *p*_3_,

where the fitted parameters *p*_1_, *p*_2_ and *p*_3_ have a direct physical meaning; 
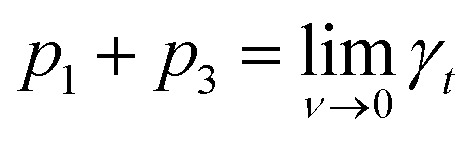
, *i.e.*, *γ*_*t*_ at low surface coverage, 
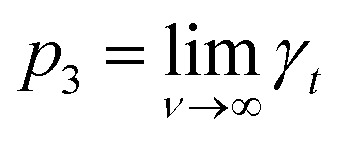
, *i.e.*, *γ*_*t*_ at full surface coverage and *p*_2_ determines how quickly *γ*_*t*_ decreases from *p*_1_ + *p*_3_ to *p*_3_ with the coverage *ν*. Hence, *p*_2_ is related to the number of high-energy sites, *i.e.*, the lower the *p*_2_, the higher the amount of high-energy sites.

### Experimental setup

Inverse gas chromatography was conducted using a surface energy analyzer SMS iGC-SEA 2000 instrument (Surface Measurement Systems, UK). The analyses were performed using 3 mm (internal diameter) silanized glass columns which are 30 cm long filled with samples (the weights and surface area are listed in Table 2). Silanized glass wool was used to plug both ends of the column containing the sample to prevent machine contamination. The injection of solvent vapors was controlled to pass a set volume of the eluent through the column to give pre-determined fractional coverage of the sample in the column. Adsorption measurements were performed using *n*-hexane (Merck, for liquid chromatography LiChrosolv®, ≥98%), *n*-heptane (Sigma Aldrich, puriss. p.a., Reag. Ph. Eur., ≥99% *n*-heptane basis (GC)), *n*-octane (Sigma Aldrich, puriss. p.a., ≥99.0% (GC)), ethylacetate (Lach-Ner, pro HPLC, min. 99.8%), and dichloromethane (Merck, for liquid chromatography LiChrosolv®, ≥99.9%). Primary chromatograms were recorded at a temperature of 323 K using He as the carrier gas at a flow rate of 10 sccm. The column temperature was controlled by using an instrument oven with a declared stability of ±0.1 °C. Retention volumes of probes were calculated from the primary chromatograms using the peak center of mass and methane was used for the dead time estimation. The measurements were repeated for various target surface coverages *ν*_i_, which ranged from 1% to 20% of the monolayer. The saturated probe vapors were injected into the column, and the injection time was set up to reach the targeted surface coverage. The required injection time was calculated from the targeted surface coverage, the known surface area of materials, adsorbate vapor tension at 50 °C, and adsorbate cross-sectional area using Cirrus Control Software advanced version 1.4.1.0 (Surface Measurement Systems Ltd, UK). The surface thermodynamic properties were calculated using Dorris & Gray and Della Volpe scale methods from the primary data using Cirrus Plus Software advanced version 1.4.1.0 (Surface Measurement Systems, Ltd, UK).

### Electrochemical measurements

Electrochemical measurements were performed on an Autolab PGSTAT204 operating in a three electrode setup. Glassy carbon (GC), platinum wire and KCl saturated Ag/AgCl electrodes were used as working, auxiliary, and reference electrodes, respectively. TMD suspensions with a concentration of 1 mg mL^–1^ were prepared by sonication for 0.5 hours in deionized water. An aliquot of 5 μl was drop-cast onto the polished GC electrode and left to dry at 70 °C for 10 minutes. GC electrodes were polished with 0.06 μm alumina slurry. After polishing, electrodes were sonicated for *ca*. 1 min in deionized water and methanol, respectively.

Heterogeneous electron transfer (HET) measurements were carried out in 1 mM solution of [Fe(CN)_6_]^4–/3–^ in 100 mM KCl. The scan rate was 100 mV s^–1^. The solution was purged with argon prior to every measurement. Measurements were repeated three times. The new portion of the tested material was drop-cast every time.

Hydrogen evolution reaction (HER) measurements were performed in 0.5 M H_2_SO_4_ solution purged with argon. The scan rate of 2 mV s^–1^ was used. Measurements were repeated three times with the new portion of the material. Exchange current densities were acquired from Tafel slope graphs by extrapolation of the linear part to zero overpotential.

### Preparation of the exfoliated samples

Exfoliation was performed in a glovebox under an inert argon atmosphere. For the MoS_2_ BuLi sample, 2 g of MoS_2_ was placed in a flask and 25 mL of 2.5 M *n*-butyllithium solution in hexane was added. The sample was stirred for seven days before filtration. After filtration, the sample was exfoliated by the addition of water under an inert atmosphere and dialyzed for several days. Finally, the sample was centrifuged and dried in a vacuum oven for 48 h prior to further use. Similarly, 2 g of MoS_2_ was used for the preparation of the MoS_2_ NaNAFT sample. 0.2 g of sodium metal, 1.2 g of naphthalene and 50 mL of tetrahydrofuran were then added and stirred for seven days. The rest of the procedure was identical to that of the MoS_2_ BuLi sample.

### Correlation tests

The correlation plot was constructed within RStudio 1.0.136 software using R version 3.3.2. The statistical significance of the correlation was tested using the test of linear independence with *H*_0_: *ρ* = 0 and test criterion 
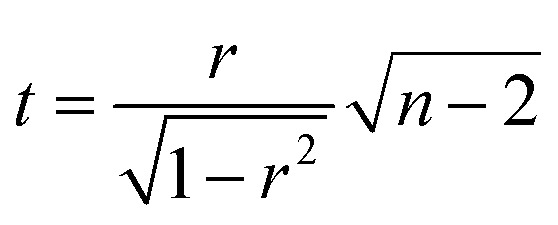
, which has Student's *t*-distribution with *n* – 2 degrees of freedom. *H*_0_ was rejected, if |*t*| > *t*_crit_ (*n* – 2).

### Scanning electron microscopy

A scanning electron microscope (SEM) Hitachi SU6600 with an accelerating voltage of 5 kV was used for obtaining SEM micrographs of MoS_2_ samples. The dry sample was placed on the support carbon grid and attached to the double-sided conductive carbon tape on an aluminum holder.

### X-ray photoelectron spectroscopy

High-resolution X-ray photoelectron spectroscopy (XPS) was performed using an ESCAProbeP spectrometer (Omicron NanoTechnology GmbH, Germany) with a monochromatic aluminum X-ray radiation source (1486.7 eV). Wide-scan surveys of all elements were performed with subsequent high-resolution scans of the Mo 3d and O 1s. Relative sensitivity factors were used to evaluate the molybdenum sulfide and oxide ratios from the survey spectra and oxygen concentration.

### Specific surface area

The surface area was measured using a sorption analyzer, Coulter SA 3100 (Beckman Coulter). The samples were outgassed for 4 hours at 200 °C under high vacuum (0.05 Pa) prior to the sorption experiments. The reason for such a low temperature is to avoid degradation and further decomposition of oxygen functionalities. A TCD nitrogen cooled (77 K) detector was used for the evaluation of the results using BET (Brunauer, Emmett and Teller) and Kelvin equations.

### Laser diffraction

The size distribution measurement was performed with a Particle Sizer Analysette 22 NanoTec (Fritsch Laborgerätebau GmbH, Idar-Oberstein, Germany) using a laser diffraction method. The measurement was performed in aqueous dispersion using a 655 nm laser.

## Conflicts of interest

There are no conflicts to declare.
